# Using a participatory action research approach to explore, understand and evaluate well-being among children living in socially deprived areas in Southern Sweden: a study protocol

**DOI:** 10.1136/bmjopen-2024-086406

**Published:** 2024-08-01

**Authors:** Rathi Ramji, Elisabeth Mangrio, Therese Sterner, Katarina Sjogren Forss, Slobodan Zdravkovic, Anders Kottorp, Louise Burenby-Yxne, Gabriella Isma, Monika Stanikowska, Julia Brandelius, Talina Marcusson Journiette, Margareta Rämgård

**Affiliations:** 1 Department of Care Science, Malmo University Faculty of Health and Society, Malmo, Sweden; 2 Malmö Institute for Studies of Migration, Diversity and Welfare, Malmö University, Malmo, Sweden; 3 Department of Culture, City of Malmo, Malmo, Sweden

**Keywords:** Community-Based Participatory Research, Community child health, Health Equity, Surveys and Questionnaires, PUBLIC HEALTH

## Abstract

**Abstract:**

**Introduction:**

Research suggests that participating in after-school leisure activities has been related to promoting health, well-being and safety among children living in disadvantaged neighbourhoods. The United Nations Child Rights Convention emphasises the inclusion of children in decisions that concern them. However, children seldom are involved in designing implementing and evaluating health promotional environments. The aim of this programme is through a participatory process with children, parents/guardians, and peer-activity leaders explore, measure and evaluate the impact on children’s overall well-being related to the social context in an already established health promotion environments in Southern Sweden.

**Methods and analysis:**

The project is based on a previously implemented unique community-based participatory research (CBPR) model for equal health in three socially disadvantaged areas in Malmö. All activity house (AAH) is a meeting place for children established in schools but after school time by the culture department of the Malmö municipality. In AAH migrant children participate in need-driven after school activities that they themselves create and develop. To increase participation of the children and ensure that these environments are based on their needs, 30 children (10–12 years), parents/guardians (30), peer-activity leaders (15), and researchers create CBPR teams in the areas and engage in a participatory process. The children reflect, analyse and write about their well-being; identify and discuss key factors in an iterative process, which also includes a strategic group of stakeholders. The children then develop and validate (with 100 other children from AAH) the Socioculturally Aligned Survey Instrument for Children survey inspired by the KIDSSCREEN V.27. The survey tool so developed will further be used to evaluate AAH and will be distributed to all children participating in their activities.

**Ethics and dissemination:**

This programme has been approved by the Swedish Ethical Review Authority. The results from this programme will be published as reports and scientific publication.

**STRENGTHS AND LIMITATIONS OF THIS STUDY**

- The research is children focused, where effects are measured through active dialogue with the children.
- The programme follows a unique model discussing, evaluating, and testing opportunities together with children.
- The research is built upon trust-based relations between the children, researchers, peer-activity leaders, parents/guardians and stakeholders.
- Children and researchers also engage in dialogues with policymakers on a strategical level.
- The strategic group shall share knowledge gained from the programme to their respective organisation, so as to initiate system change.

Investing in children’s overall well-being lays foundation for healthy behaviours and educational attainment with impacts that resonate even into adulthood.^1^ The United Nations convention states all children have the right to the highest attainable standard of health and well-being. Children’s well-being is rooted within the social and environmental determinants surrounding them particularly in relation to their home and school.^2^ However, poverty is one of the factors that has contributed to growing inequalities in children’s health and well-being.^3^ This seems even more true particularly among children and youth living in disadvantaged neighbourhoods. These children are more exposed to risk behaviours such as because of lower socioeconomic status, poverty, migration status as well as crime in the local environment.^4 5^

To promote well-being among children, it is important to enhance their ability to make healthy decisions, which may have a significant influence on their overall health and well-being in the long term. Health promotion interventions should aim to achieve overall well-being and eliminate health disparities. Participation in organised leisure-time activities has been identified as a means to enhance development, promote health and well-being and increases a sense of safety among children particularly in vulnerable contexts.^5^ Participation in leisure activities after school can give children the opportunity to fulfil their health needs and conditions to experience an increased sense of connection.^3^ Involvement in different organised activities has also been associated with increased academic achievements, social relationships, life satisfaction and better mental health.^6 7^ Being together with friends or peers in different social activities is of great importance for children’s overall well-being.^8^ The presence of a local context or meeting places in near proximity has been pointed out as the strongest predictor of active participation in sports or other activities with friends and peers.^5^

The school environment has been regarded as an ideal location for promoting health, since children spend most of their time there and also since schools are geographically easily accessible.^9^ The WHO also reports that health promotion programmes held in the school premises can reduce behavioural problems as well as influence positive health and well-being.^10^ Although participation in leisure time activities after school has been regarded as health promoting,^11^ there is a need to understand the impact of the activities on their well-being and to evaluate the sustainability of activities based on their perceived needs. To better understand children’s own perspective, their active participation is important in such efforts. The Child Convention also articulates that children must be given the opportunity to express their views on issues that concern their health and well-being.^3^ Although there exist school-based health promotional programmes^12^ where children are actively involved in the research processes, participatory health promotional initiatives facilitated by stakeholder outside the school organisation are relatively uncommon

Children especially from migrant backgrounds are often regarded as passive instead of being active social actors when it concerns decisions regarding their well-being.^2^ The children’s perspective as an agent in a situation represents how they experience perceive and understand a context in relation to themselves and their context. Drawing on a strength-based approach where focus is on the personal strengths, social and community networks rather than their deficits empowers and actively engage children to take more part in the research process. This approach emerges from the belief that children possess invaluable knowledge and skills that contribute positively to the research process and the outcomes.^13^

Thus, the overall aim of this programme is through a participatory process with children, parents/guardians and peer-activity leaders explore, measure and evaluate the impact on children’s overall well-being related to the social context in already established health promotion environments in three socially vulnerable city areas in Southern Sweden.

## Description of AAH intervention programme

The Leisure and Culture department in the municipality of Malmo started health promoting environments known as all activity house (AAH) with after school activities for children and youth based in the school premises in the areas within the cities where families have lower socioeconomical circumstances and higher child poverty then the rest of the city. The activities within this programme are themselves participatory in that they are based on the children’s own needs. The activities are designed in continuous dialogue and contact with the children and families in the geographical community where the schools are located. The families also participate in some of the activities. Prior studies suggest that children living in these areas in Sweden are seldom able to participate in the states regular leisure activities since their parents (often the mother of the child) is not working. The state organised activities are intended for children with working parents. The activities in the AAH on the other hand are open to all, completely free of charge and happen all through the year even during school holidays. The AAH has also become a meeting place for the families and organisations in the communities. Over time, the AAH has become important places in the children’s life, and they have developed a unique organisation where young leaders work with the children in a peer support system within the different houses.^14^

AAHs were established in year 2011 by municipality of Malmo (Malmö stad). Currently, there are four AAHs in collaboration with elementary and middle schools located in Lindängen, Rosengård, Hermosdal and Helenholm. The municipality also intends to establish several new AAH in similar neighbourhoods in the near future. The AAH although physically located in the schools is independent of the school organisations that conduct about 60–80 activities per day. During year 2023, 81 970 persons visited the activities of which about 3% were over 18 years. Both boys and girls participate in the activities within AAH. In year 2023, nearly 40 151 girls/women and 41 819 boys/men were regularly engaged with AAH.

The AAH house is regarded as a platform that creates the opportunity to try various forms of physical activity such as football, boxing, dance to healthy diet (cooking and baking). The activities are suggested by the children themselves and then facilitated and coordinated by the peer-activity leaders. These activities also tend to focus on the children’s well-being while also promoting health. The activities were available on all days of the week, including weekends. Alongside with the activities, the programme also offers breakfast at schools. In addition to the activities, the peer-activity leaders also engage discussions on important topics such as safety, children rights and equality with the children. Earlier research has shown that facilitating health promotion activities in vulnerable neighbourhoods predominated by migrants in collaboration with local stakeholders, and the children can increase social cohesion and contribute to children’s well-being and resilience.^15 16^

Research projects related to this kind of programme are rare in Sweden but could be of interest to the international research community contributing to how children and families can take an active part in building resilience through child-driven participatory action research (PAR) processes. One way to approach community resilience from this view is using participatory approaches such as community-based participatory research (CBPR),^17^ where children are active participants in research. In addition, children’s health promotion activities should be followed up where the children themselves participate in the processes and the evaluation of programmes. Using a CBPR approach together with the children can link the voices of children, and their families with local stakeholders in a process of building resilience among them.

## Theoretical framework

A holistic approach to children’s well-being includes physical, mental, emotional and social and environmental aspects.^18^ Health promotion is regarded as an important tool for improving health and reducing costs for national health systems. It can be seen as a process to enable people to take control over and improve their lives.^17^ It is important to ensure that a health promotion initiative encompasses all these components, it may not be enough to focus only on the physical, mental and behavioural aspects (such as physical activity and nutrition). An effective health promotion initiative should also promote building elements such as emotional intelligence, resilience, social cohesion, environmental awareness, creativity and play.^19^

Furthermore, health promotion must empower and match the varying needs and expectations of the people it is oriented towards as stated by the Jakarta declaration.^20^ A top-down centralised approach in health promotion activities may neglect the positive role that children/youth in local communities can play in a health promotion programme that aims to strengthen social cohesion. This is in particular a challenge where local knowledge, cultural nuances and community needs are in play.^21^ The WHO has also identified that there is a need for local solutions to tackle the root causes of social inequities, where communities and individuals can engage as important partners in cocreating solutions.^20^ Cultivating resilience through health promotion activities together with children, their parents/guardians and peer-activity leaders could provide knowledge regarding risk-reducing mechanisms for migrant children living in these disadvantaged environments.^16^ It is often described in research that inequality and social disparities within and across these socially vulnerable communities may lead to poor health, but it is also important to take into account that community assets such as solidarity, mutual trust and strong social networks in health promotion environments are recognised as protective factors that can promote positive health trajectories and social cohesion for children.^22^ The United Nations ratified a convention on the Rights of Children, which insists that children are also individuals with their own rights.^23^ Sweden was one of the first countries to ratify the convention already in 1990 (SOU 2016:19), and in year 2020, it was reinstated as an official law. Thus, Swedish public sector and its employees must both respect, protect and comply with the convention and do their part towards child equality and rights. To strengthen children’s rights, it is necessary to give children the opportunity to express their views on issues that concern them. One way to approach health promotion from this view is using participatory approaches such as CBPR, where children are active participants in research.

The theoretical approach behind CBPR this research is part of an emancipatory, reflexive and critical social scientific tradition,^11 12^ in that the participants together, and in collaboration with the researchers, ‘attempt to remake and improve their own practice to overcome distortions, incoherence, contradictions and injustices’ through collaborative dialogues and cocreation actions in the framework of PAR.^13^ Paulo Freire (1972) defines dialogues ‘as a meeting between people in order to “name” the world, a fundamental requirement for their true humanization’.^14^ According to Kemmis and McTaggart, reflexive dialogues are related to social cohesion because it ‘engages participants in a collaborative process of social transformation’.^13^ We expect that the collaborative activities in this proposal will function as communicative spaces for such dialogues from the children’s perspective. From a communicative perspective, these ‘pedagogical encounters’ have similarities to Habermas’ idea of an *ideal speech situation*^15^ (p86 ff), a situation in which all people involved have an equal right to participate in the dialogue as well as expressing ‘attitudes, desires and needs’,^15^ and thereby also influence decisions. These kinds of dialogues are an essential part of the participatory research process.

Kurt Lewin (1941) coined the term action research, which can be described as an organisational change theory that bridges practice and theory gaps through action research cycles with a pragmatic approach. This approach contrasts to a more emancipatory approach of Freire where researchers join a social movement and challenge dominant knowledge cultures inside academia.^14^ However, today many partners integrate both these approaches in their research.^16^ Participatory research as a research paradigm means that participation is the defining principle throughout the research process. For PAR, the primary underlying assumption is that participation of those whose lives or work is the subject of the study fundamentally affects all aspects of the research and involve an action in practice. The engagement of these people in the study is an end in itself and is the hallmark of PAR, recognising the value of each person’s contribution to the cocreation of knowledge in a process that is not only practical but also collaborative and empowering.^17 18^ As the most well-known community-engaged research approach within public health, health promotion and health sciences, CBPR extends PAR beyond ‘shared leadership’, to ‘community-driven’ approaches, where children/youth in the communities decide research priorities, participate in all research steps and complement actions from findings.^17 19^ This approach also has a clearer framework in the name of Paulo Freire (1970) arguing that reflection/action/ reflection cycles of knowledge creation can foster democratic participation of citizens in the community towards social transformation. CBPR partnerships can be defined as a ‘collaborative effort between community, academic and other stakeholders who gather and use research and data, build on community citizen strengths and priorities and adopt multilevel strategies to improve health and social equity’.^19^

Although several CBPR studies often focus on health-promoting activities among children, the CBPR approach is frequently applied at the organisational level relating to the communication between the academia, school officials and even adults from the community but seldom children^12^ especially in the Northern Europe . CBPR projects seem more common among adults than children owing to the misbelief that children’s cognitive skills are not developed enough to understand the research process. It is important that children also are involved in the research processes.

There is a growing body of research that involves children in visual methods and art-based methods.^13^ But as Coyne and Carter argue there is a lack of understanding on the degree of partnership between the children and the research team owing to poorly described methods and also that the participatory processes are not clear.^13^ Research also suggests that coresearching with children leads to more effective and responsive practices that influence their well-being and development.^12^

It is important that the participatory research can contribute to sustainable interventions and evaluations in health promotion. Most of the existing health promotion activity programmes had a predetermined agenda defined by adults to address specific issues such as obesity or prevention of violence and were offered as a short-term educational courses.^24^ Having a long-term perspective in mind that children need to acclimate to responsible citizenship and good decision-making skills taking into consideration the social determinants of health, they must be given opportunities to develop participatory skills during their early years. Equal distribution of power in research particularly when working with research related to children’s well-being is important.^25^ Combining qualitative and quantitative method in the PAR processes will make the CBPR interventions more sustainable over time.^26^

Therefore, this research programme has several CBPR projects, so that the participatory processes are integrated in the community practice in all of the AAH. Coresearch with the children is expected to result in relevant and meaningful insights that directly inform practice and facilitate the evaluation of the AAH intervention.

## Methods and analysis: the research programme

**Figure 1:**
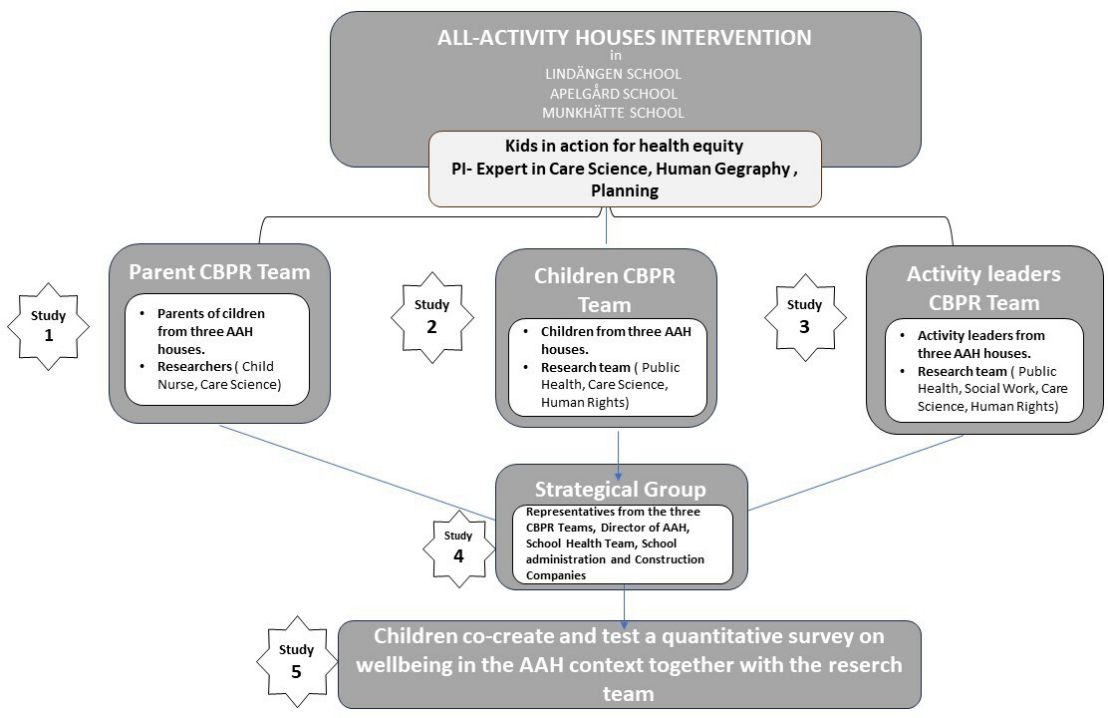
Overview of the research programme Kids in Action promoting health equity. AAH, all activity house; CBPR, community-based participatory research.

The research programme involving the AAH will be facilitated by researchers from Malmö University, in collaboration with Malmo City (municipality of Malmo) and the geographical communities participating in AAH houses at the schools in Rosengård (Åpelgård school), Lindängen (lindängen school) and Helenholm (Munkhätte school). The research applies a participatory research design with elements of both qualitative and quantitative approaches. The programme includes five distinct projects focused on children’s well-being. The Research Programme had a start date 1 December 2022 and shall happen until 30 November 2027. The research group has since start established contact with AAHs, secured ethical approval and engaged in trust-building activities for a prolonged period and the empirical research September 2023. Studies I, II and III are currently ongoing (parallelly) where different researchers from the research group are engaged in the three CBPR teams. The overview of this research programme is presented in figure 1.

### Project I—CBPR team—children

#### Participants

Children aged 10–12 years (middle school age) are to be included as evidence suggests that children aged 10 years and above had the verbal competence and extroversion level necessary to participate as equal partners in researchers. In addition, prior research also shows that children in these age groups are often exposed to negative social elements in the society.^27^ Children shall be recruited from all the three AAHs. The researchers will not take part in the recruitment process but will hold a meeting in each of the AAHs to inform the peer-activity leaders and managers about the research project and the CBPR team’s work. Written information about the project will be distributed through the AAHs to the children and their parents/guardians (see table 1). Participation is voluntary, parents/guardians and children shall contact the AAH and express their interest to participate.

**Table 1:** Characteristics of participants in the different studies within this programme

Participants	Gender (n)	Location—school (n)	Total participants
**CBPR teams*Children (age 10–12)***	Girl (15)Boy (15)	Rosengard—Apelgård school (10), Lindängen—Lindängen school (10), Helenholm— Munkhätte school (10)	30
***Parents/guardians***	Male (15)Female (15)	Rosengard—Apelgård school (10), Lindängen—Lindängen school (10), Helenholm— Munkhätte school (10)	30
***Peer-activity leaders***	Male (2)Female (1)	Rosengard—Apelgård school (5), Lindängen—Lindängen school (5), Helenholm— Munkhätte school (5))	15
**Steering group*Children (age 10–12)***	Girls (8)Boys (7)	Rosengard—Apelgård school (5), Lindängen—Lindängen school (5), Helenholm— Munkhätte school (5)	15
***Parents/guardians***	Male (3)Female (3)	Rosengard—Apelgård school (2), Lindängen—Lindängen school (2), Helenholm— Munkhätte school (2)	6
***Director for localall activity house(AAH)***	Male	Rosengard—Apelgård school (10), Lindängen—Lindängen school (10), Helenholm—Munkhätte school (10)	3
***Executive director***		Malmö city	1
***Developing coordinators for children’s rights***	Male (1)Female (2)	Rosengard, Lindängen, Helenholm	3
***Property owner***		Rosengard, Lindängen, Helenholm	3
***School health team***	Female (3)	Schools in Rosengard Lindängen Helenholm (Munkhatte)	3

CBPRcommunity-based participatory research

Children and parents/guardians who consent participation will be included in this project. To increase participation of the children and ensure that these environments are based on their needs, 30 children (10–12 years) together with researchers create a CBPR team in each of the three AAHs. The research team will ensure the inclusion of equal numbers of boys and girls. All children included in the study are expected to communicate in Swedish language.

#### Participatory methods and procedures

**Figure 2:**
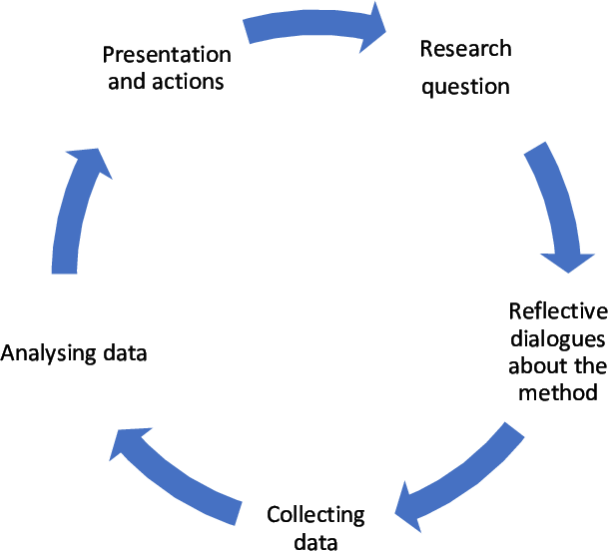
The action research cycle that will happen in each CBPR team. CBPR, community-based participatory research.

The aim of this project is to define children’s overall well-being and the key issues in their social context that influences their overall well-being together with them. The CBPR teams are expected to meet on several occasions within this project during a year. The children shall be encouraged to reflect, analyse and write about their well-being; identify and discuss key factors. The researchers facilitate a process where the children are actively engaged in defining aim, designing the research process and analysing the material. This project is a participatory research design with a qualitative approach using multistage focus (MSF) groups.^28^ MSF groups were deliberately chosen as it suits well given that through engaging children in series of meetings in regular intervals may facilitate the process of intertwining research with action. This approach may allow action and critical reflection to occur consecutively in a cyclic or spiral manner, ensuring that each stage of the research builds on the preceding moment and is informed by ongoing reflection and feedback (see figure 2).

Each focus group meeting will be planned together with the children and the AAH personal, and the activities will revolve around the central theme namely well-being starting from exploring what well-being in the context of AAH mean for the children. The children will in the first cycle (a) discuss the meaning of well-being, (b) have reflective dialogues about the research questions relating to the method, (c) take action by collecting data through photos representing their individual definition of well-being, (d) analyse data in a thematic analysis in the focus group, (d) present the results in form of a photo collage to the group in the AAH and their parents/guardians. The second circle will be followed by a deeper exploration of factors associated with well-being, so as to answer the question ‘why’ to understand causes, consequences and constraints towards achieving well-being through (a) discussing the second research question, (b) having reflective dialogues about the second research question relating to the method, (c) create a model of their photos with explanations, (c) analysing data through the Bronfenbrenner model in the focus group, (d) present the results through a photo collage to the group and the AAH to suggest further actions.

The third circle will focus on action to answer the question ‘now what’ where key action points will be identified and also how the information can be used or presented to stakeholders and decision-makers together with the children.

Before the research starts, the members of the research team will engage in trust building and team building activities through organising activities such as quizzes to familiarise the overarching goals of the research and also team sport competitions to enable a better relation between the research team and the children as well as among the children themselves. As it may be challenging to directly engage children in discussions, and dialogue within focus groups, photovoice method shall be used as it is useful to describe complex concepts such as resilience. In this project, the method shall be used to initiate and reflect on aspects such as well-being and the social context, they encounter in the AAH. In the following action circle, the children will reflect again on their photographs with the group and may also add some new after the dialogues with the other children.

Following this, they will then work with the second research question: factors relating to their well-being within their immediate as well as extended environments and reflect on that. The analysis of this actions will be inspired by the Urie Bronfenbrenner’s socioecological model. The model focuses on the individual as a person, the nature of interactions (process), the surrounding environment (context in which the individual thrives) and the influence of these factors over time. During this session, each child will place their pictures that symbolise factors related to their well-being considering at what level of importance these factors are in their life in a chart with excentric circles at four levels, namely individual, mesosystem, macrosystem or exosystem. Field notes and audio recordings are to be made during this session. Results are later written by the researchers following discussion with the children about the written text. Consecutively, during the next session, all the children shall be engaged in the AAH steering group meetings where the children together with leaders shall discuss further steps regarding what changes in their near environment may further improve their well-being in the communities

These action points will further be presented by the children together with the research team to relevant stakeholders in the next stage of the programme (project IV).

### Project II—CBPR team—parents/guardians

#### Participants

The CBPR team will include parents/guardians of children participating in all of the AAH and researchers. Written information about the project will be distributed through the AAH staff. Participation is voluntary, parents/guardians who are interested are expected to contact the research team and express their interest to participate. The research team will ensure that there is a balance of both women and men. If parents/guardians do no’t understand or speak Swedish or English interpreters from the AAH will be used during the project.

#### Participatory methods and procedures

The aim of this project is to define the parent’s/guardian’s views on children’s overall well-being and the key issues in the social context that influences children’s overall well-being. Data will be collected through MSF groups with 8–10 participants on two to three occasions, where the CBPR teams reflect and discuss about a predetermined topic.^28^ The use of MSF groups is a method where participants have something in common (age, gender, experience, etc), and the question time is led by interviewers/moderators from the research team who are a part of the CBPTR team. Researchers facilitate the reflective dialogues in the group conversation, and the perspective on the subject is broadened, so that the participants can express their opinions while the others listen and replenish with their own experiences. In such a focus group, the participants will talk about their own opinions while listening to others and supplementing their experiences in different participatory dialogues. The interaction between the participants provides a great depth in the answers to the questions that are illuminated from a variety of perspectives, despite few participants. At the end of the meeting, the moderator summarises the current conversation and conclusions into actions. In the next focus group, the moderator starts repeating the summary of these actions and then continues with new questions around the overall theme. Following an understanding of children’s well-being from the parents’ perspective, the researchers also facilitate discussion regarding how the parents can further assist in improving well-being among children and what support they may require from the AAH to be able to improve their children’s well-being in the consecutive sessions. Actions points agreed mutually with the parents that are taken forward to the strategic group meeting with stakeholders in project IV.

### Project III—CBPR team—peer-activity leaders

#### Participants

The CBPR team will include peer-activity leaders working in all of the AAH and researchers. All peer-activity leaders with at least a year of work experience in AAH shall be invited to participate in this study. Written and verbal information shall be provided ensuring that participation is voluntary. Peer-activity leaders who accept to participate shall also be asked to provide written consent.

#### Participatory methods and procedures

Dialogues were held in MSF groups^28^ with the peer-activity leaders in the CBPR team. Between the focus group sessions, the peer-activity leaders and the research team shall, in engage, participate in the activities facilitated by the leaders in the AAHs. The aim of this study is to define the children’s well-being and the key issues in the social context that influences children’s overall well-being together with the peer-activity leaders in their work with the children.

The peer-activity leaders will in the first cycle (a) discuss the aim, (b) participate in reflective dialogues in the focus group, (c) engage in the activities with the children in the AAH, (d) reflect on the experiences followed by analysing and creating themes based on the reflection with the researchers.

The reflections in the focus groups shall focus on the peer-activity leaders’ views regarding the children’s well-being. The leaders together with the researchers shall also be engaged in thematisation of the key insights from their discussions, which is then summarised by the research team. During a consecutive session, the leaders and the researchers also discuss action points that may involve what and how they can further contribute to the well-being of the children participating in AAH and what resources they may need for this. Key action points from these discussions will also be presented and discussed in the strategic group meeting in Project IV.

### Project IV

#### Participants

In this project, the results from the three initial projects shall be discussed in an iterative process between strategic group of stakeholders, children, parents/guardians and peer-activity leaders and the CBPR teams. The strategical group shall include stakeholders from the school’s health team (student health), the municipality (personnel in AAH) and representatives from property owners and state representatives from the County Council of Scania. Each of the AAH has earlier collaboration with property owners and health teams in the different schools as part of their daily work.

#### Participatory methods and procedures

Knowledge from the CBPR teams in the first three projects will be transferred by the research team through a series of reflective dialogues with the stakeholders from the strategical group to deliberate further, reflect and discuss the findings between focus group meetings described above. The dialogues will be held in two multistakeholder workshops where a qualitative research method namely dialogue café^29^ will be used. In this method groups of six to seven participants are seated in café-style tables and will engage in dialogues. All groups will have a representation from children from all three AAH houses who will engage in discussions with stakeholders in a power neutral environment. At least three consecutive rounds of dialogue, where each round lasting for approximately 15 min, take place. The same question or idea is discussed by the participants in each table. Participants rotate around the tables at the end of each round, moving from one table to a new one that they have not earlier visited, and the discussions continue with a new group of participants in that group. Each table has a host/facilitator who remains in the same position throughout to collect and summarise the essence of the reflections at that table. It is then collected by the responsible researcher who participates in the workshop. This method is useful when aiming for a democratic voice instead of certain people alone dominating the conversation.^30^ The dialogues in the focus groups will be recorded, transcribed by the research team.

**Figure 3:**
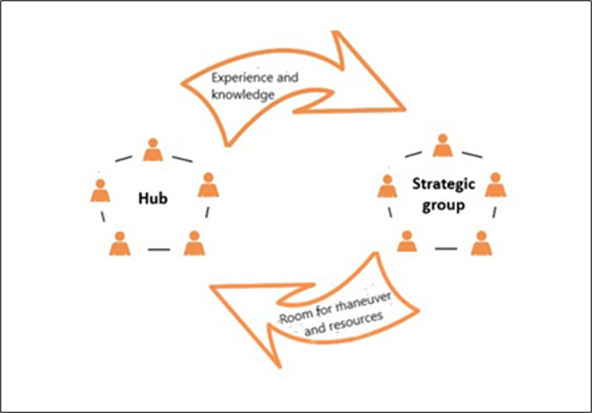
Knowledge transfer between the hub and strategy group.

The processes in the data collection above can be described at two levels ‘from below’ and ‘from above’ finding each other and hooking each other during the process. During such a process, knowledge from different sources is mobilised.^31^ Each phase may need to ‘loop back and forward’ a few laps between CBPR teams and the strategic group, to ensure that a sufficiently resilient foundation has been created and that the work process can enter the next phase. The strategic group should above all be understood as a group for learning where ‘new knowledge’ can arise through the group’s reflections (see figure 3), through the joint learning processes in the group, and by the group members creating room for manoeuvre and resources for changed action in their organisation (AAH). These loops contribute to the description of the needs that exist among the children and their parents/guardians becoming a real input for understanding how the actual system works in relation to various initiatives in the AAH. For the project’s function, it is crucial that all participating partners have an active and cocreative role in the joint work and have developed channels at a strategic level in their own organisation to take advantage of the learning that takes place within this process. It is only then the project’s system-changing ambition has the opportunity to be realised. A large and important part of the learning that the project enables is, therefore, also about the municipality increasing its understanding of how its own organisation can work in a more effective way to achieve its own organisational goals-through policy changes and equal health in society for children.

The programme will aim at bringing out action points that change the context to fit the children’s needs and shall be followed up by the children through actively engaging in the AAHs organisation as a reference group. They will also develop an instrument and follow-up the changes longitudinally

### Project V—development and validation of SASIC

The results of the coresearch with the children which after being discussed in the strategic group shall also be further explored to seek ideas on the structure, content and structure for an evaluation tool on overall well-being. Three workshops are to be held to engage the children in the development of the instrument Socioculturally Aligned Survey Instrument for Children (SASIC). The purpose of this survey tool is to capture and potentially measure children’s perceptions of the activities they participated in and their impact on their overall and well-being longitudinally. Within CBPR, the process of developing and validating an instrument is a non-linear process that demands extensive communication, active listening and consideration of preferences from multiple sources.

#### Participants

Children from the AAH who participated in the first project shall be contacted through the peer-activity leaders and asked to participate in this study. The researchers will inform the children and their parents/guardians (via a written information letter) about the aim of this study and the different workshops the children shall participate. Participation is voluntary, children are included in the study after obtaining consent from the children and their parents/guardians.

#### Participatory process in the development of an instrument

Three workshops will be held to identify key domains based on the thematic analysis of subjective and explorative discussions from the CBPR teams are identified together with the children. Furthermore, the key domains the children themselves highlighted shall be compared with the themes included in a previously validated national surveys for school-aged children.^32 33^ Finally, questions shall be formulated under each of the identified domains taking inspiration from other validated tools. The questionnaire developed following the three workshops will then be presented and discussed with the children, who will themselves present it to the strategical group where representatives from different stakeholders give their input. The children who participated in the CBPR teams will become a reference group within the AAHs organisation and will be part of further testing, discussing and monitoring the use of this survey as well as the results.

The questionnaire shall initially be developed in Swedish language as it is the operating language of the AAH. Following this stage, the SASIC will be evaluated based on test content by 30 children aged 10–12 years from all three AAH, but who have not been part of its development. The questionnaire shall be translated to other languages in future, so as to enable representativeness and a higher degree of participation among children from non-Swedish backgrounds following discussion with the reference group.

A Think Aloud Method in combination with observations will be used to discuss the questions and response options within the newly developed questionnaire with a focus on four aspects including experiences on concurrent aspects of the questions, perceptions related to retrospectively recalling their experiences, concerns regarding sensitive questions or problematic phrases or words. The results of these interviews are to be analysed to understand the challenges and strengths of the survey. A constant comparison approach is to be applied in an attempt to recognise patterns in children’s responses. The results of the think-aloud study and any suggested changes that may appear will then be presented and discussed with the children in the reference group who participated in the development of questionnaire.

The revised questionnaire is to be pilot tested among a selected group of approximately 100 children between 10 and 12 years from all the three houses. The research team together with the reference group who initially were engaged in developing the survey shall reach out to other children while they are engaged in the different activities in AAH and shall inform both verbally and in written form about this study. The study will aim to include a representative group of children from the different houses in terms of gender and age. Consents from the children and parents/guardians are obtained ahead of distributing the surveys. Responses from the surveys will be examined in relation to validity in response processes and internal structure using both classical and modern test theory approaches.^34 35^ The measures with the revised SASIC questionnaire codeveloped by the children shall be compared with the KIDSCREEN V.27 to explore if they measure potentially similar constructs.

The revised version of the validated SASIC survey shall be distributed to all children participating in the AAH between ages 10 and 12 years with the support of the peer-activity leaders and the AAH personnel in close collaboration with the reference group with an aim of longitudinally evaluating the AAHs.

### Patient and public involvement

Patients and/or the public (children) were involved in the design, or conduct, or reporting or dissemination plans of this research. Refer to the manuscript as a whole, it is built on CBPR principles.

## Ethics and dissemination

The Swedish Ethical Review Authority has approved this research programme (DNR 2023-00979-01). Participation in the study is voluntary. All participants including the children, parents/guardians and peer-activity leaders shall be given verbal and written information about the study, including details of what their participation entails ahead of requesting informed consent.

According to Swedish laws, both children and parents/guardians should consent for children’s participation. Therefore, the children were also requested to consent to participation. In addition, the parents/guardians of the children who shall be part of the Children CBPR team shall also be provided information regarding their children’s role in the programme and shall be requested to sign the informed consent forms consenting for their children’s participation in this programme. Although this programme primary focuses on children’s well-being, parents/guardians of the children are also participants and shall be asked to provide separate written consent to participate in the parents/guardians CBPR team.

Furthermore, the research team is aware that this project involves research with a group which may be regarded as vulnerable. The AAHs personnel shall provide support to the children if any sensitive discussions emerge in the group that makes the children uncomfortable. Between the focus groups, parents/guardians will also be contacted to ensure that the children are comfortable in their respective CBPR teams. One of the advantages of the studies in this programme is that it is based on an everyday environment familiar to the children, namely the AAHs, which is located within their school premises. All data collected shall be discussed and reflected on within the CBPR teams both after the analysis is completed and before publication.

The results from this programme will be presented in: Reports to the Malmö City (municipality), a PhD thesis with international publications in peer-review public health journals, and at least two publications from the research team possibly coauthored by stakeholders and participants shall be published in high impact peer-reviewed journals.
