# Supplementary material for: Using a participatory action research approach to explore, understand and evaluate well-being among children living in socially deprived areas in Southern Sweden: a study protocol
Source: BMJ Open. 2024 Aug 1;14(8):e086406. doi: 10.1136/bmjopen-2024-086406 (PMC11298750; doi:10.1136/bmjopen-2024-086406)
Supplement: Supplementary file 1 [file bmjopen-2024-086406.reviewer_comments.pdf]

PEER REVIEW HISTORY

BMJ Open publishes all reviews undertaken for accepted manuscripts. Reviewers are asked to complete a checklist review form (**http://bmjopen.bmj.com/site/about/resources/checklist.pdf**) and are provided with free text boxes to elaborate on their assessment. These free text comments are reproduced below.

ARTICLE DETAILS

**TITLE (PROVISIONAL)**	Using a participatory action research approach to explore, understand, and evaluate wellbeing among children living in socially deprived areas in Southern Sweden – A Study Protocol
**AUTHORS**	Ramji, Rathi; Mangrio, Elisabeth; Sterner, Therese; Forss, Katarina Sjogren; Zdravkovic, Slobodan; Kottorp, Anders; Burenby-Yxne, Louise; Isma, Gabriella; Stanikowska, Monika; Brandelius, Julia; Journiette, Talina; Rämgård, Margareta

VERSION 1 – REVIEW

**REVIEWER**	Di Monaco, Roberto<br>University of Turin
**REVIEW RETURNED**	08-Apr-2024

**GENERAL COMMENTS**	The study proposes a path and a method to improve the monitoring and evaluation of All Activities house (AAH) regarding children's health. The aim is to improve knowledge on health inequalities and the effect of social determinants of health, to set up appropriate actions and policies to reduce them. The chosen method is inspired by action research, where the interested population becomes the protagonist of both the generation of knowledge and the actions to change reality. This choice is original and appropriate, especially because children are rarely considered responsible social actors and not enough importance is given to the social environment in which they live. This involvement is essential to understand and change health-related behaviors. The study design for the improvement of observation tools is consistent with this logic and well documented. However, the study should better clarify its boundaries and coherence compared to the research method. To remain consistent with the method, the observation tool, built with the active contribution of children and parents, should in turn be used in each AAH as a basis for the planning and evaluation of health improvement actions, within the scope of performed activities. Children should also be involved in the transformative phase and in the subsequent evaluation cycle, always using the tool adapted to their size and with appropriate methods. The role of the leaders of the activities in the AAH and of the strategic group should be that of facilitators of the change action, making resources and coherent decisions available. This process of involvement is what happens in all innovation development paths in organizations, where the circuit of knowledge creation and the circuit of transformation of behaviors and practices are closely connected and are part of a single process of change (lean , agile, etc.).In the proposed study this does not seem to happen, because the active involvement of the children is limited to the objective of preparing the new observation tool, while the transformation seems to be delegated to the strategic group. To improve the coherence of the study one of the following two choices

	should be adopted:1) limit the objective of the study to the preparation of the new instrument, with the active involvement of children and parents, without referring to the action research paradigm. The transformations will be managed in a traditional way by decision makers, using the knowledge deriving from the surveys with the new tool.2) Maintain the current approach of the study (absolutely appropriate) centered on action research, but modify the role of children and parents and of leaders and decision-makers in the transformation process, proposing integrated methods of planning, management and evaluation of changes to reduce the health inequalities, starting from the activities carried out in the AAH and from the change in individual and social behaviors of children, families and leaders.

**REVIEWER**	Barfield, Patricia<br>Oregon Health & Science University, Nursing
**REVIEW RETURNED**	24-Apr-2024

**GENERAL COMMENTS**	I appreciate your idea and conceptualization of this mixed-methods CBPR study. I am unclear why you provide an interpretor for adult participants but not for the children, especially since you promote childrens rights. I question if a 10-12 year old can provide consent (assent?). I did not see your plan for recruitment or data analysis.

VERSION 1 – AUTHOR RESPONSE

Reviewer: 1 - Dr. Roberto Di Monaco, University of Turin

Comments to the Author:
The study proposes a path and a method to improve the monitoring and evaluation of All Activities house (AAH) regarding children's health. The aim is to improve knowledge on health inequalities and the effect of social determinants of health, to set up appropriate actions and policies to reduce them. The chosen method is inspired by action research, where the interested population becomes the protagonist of both the generation of knowledge and the actions to change reality. This choice is original and appropriate, especially because children are rarely considered responsible social actors and not enough importance is given to the social environment in which they live. This involvement is essential to understand and change health-related behaviors. The study design for the improvement of observtion tools is consistent with this logic and well documented. However, the study should better clarify its boundaries and coherence compared to the research method. To remain consistent with the method, the observation tool, built with the active contribution of children and parents, should in turn be used in each AAH as a basis for the planning and evaluation of health improvement actions, within the scope of performed activities. Children should also be involved in the transformative phase and in the subsequent evaluation cycle, always using the tool adapted to their size and with appropriate methods. The role of the leaders of the activities in the AAH and of the strategic group should be that of facilitators of the change action, making resources and coherent decisions available. This process of involvement is what happens in all innovation development paths in organizations, where the circuit of knowledge creation and the circuit of transformation of behaviors and practices are closely connected and are part of a single process of change (lean , agile, etc.).In the proposed study this does not seem to happen, because the active involvement of the children is limited to the objective of preparing the new observation tool, while the transformation seems to be delegated to the strategic group. To improve the coherence of the study one of the following two choices should be adopted:1) limit the objective of the study to the preparation of the new instrument, with the active involvement of children and parents, without referring to the action research paradigm. The transformations will be managed in a traditional way by decision makers, using the knowledge

deriving from the surveys with the new tool.2) Maintain the current approach of the study (absolutely appropriate) centered on action research, but modify the role of children and parents and of leaders and decision-makers in the transformation process, proposing integrated methods of planning, management and evaluation of changes to reduce the health inequalities, starting from the activities carried out in the AAH and from the change in individual and social behaviors of children, families and leaders

Thank you for the valuable suggestions and thoughts. However, the research team believes that this is a PAR program involving children throughout the research process and even beyond. We do agree that the text does not sufficiently clarify how the multistage focus groups will be facilitated and how the children shall be involved throughout the research process within the program and how they shall also be integrated within the AAH organization as a reference group. This has been clarified better both in text and using a figure **(Figure 2)** in the revised manuscript on p. 8-13

Reviewer: 2 - Dr. Patricia Barfield, Oregon Health & Science University

Comments to the Author:
I appreciate your idea and conceptualization of this mixed-methods CBPR study.

The authors would like to thank the reviewer for their interest in this manuscript and the encouraging words.

I am unclear why you provide an interpretor for adult participants but not for the children, especially since you promote childrens rights.

All children in Swedish schools communicate considerably in Swedish language as it is the main language of instruction in the schools. Further, the inclusion criteria for children in this program was that the children spoke Swedish. Such an inclusion criteria is proposed as it is methodologically challenging to conduct focus groups where children speak different languages as it is very time consuming considering the need to translate from these languages to Swedish and the other way around. Prior research also suggests that one of the critical factors to consider while doing participatory action research with children is that they can concentrate or focus on the discussions for a shorter time span compared to adults [28]. Therefore, having children speaking a common language in a group can be more practically feasible and has even shown to be effective even in previous studies conducted by the research team [15, 16].

This information is also included in the revised manuscript on p.14

I question if a 10-12 year old can provide consent (assent?).

According to Swedish laws both children and parents should consent for children's participation. Therefore, the children were also requested to consent to participation. In addition, the parents have been provided information regarding their children's role in the program and are requested to sign the informed consent forms consenting for their children's participation in this program.

This information is explained more in detail in the revised manuscript on p. 7-8

I did not see your plan for recruitment or data analysis.

Thank you for bringing this up. More information on recruitment and data analysis is now included in the revised manuscript on p. 7

VERSION 2 – REVIEW

**REVIEWER**	Di Monaco, Roberto<br>University of Turin
**REVIEW RETURNED**	09-Jul-2024

**GENERAL COMMENTS**	I appreciate the additions and think they have been adequate to improve the article
